# Identification of a Novel *Afipia* Species Isolated from an Indian Flying Fox

**DOI:** 10.1371/journal.pone.0121274

**Published:** 2015-04-15

**Authors:** Brad S. Pickering, Shaun Tyler, Greg Smith, Lynn Burton, Mingyi Li, André Dallaire, Hana Weingartl

**Affiliations:** 1 National Centre for Foreign Animal Disease, Canadian Food Inspection Agency, Winnipeg, Canada; 2 Department of Medical Microbiology, University of Manitoba, Winnipeg, Canada; 3 National Microbiology Laboratory, Public Health Agency of Canada, Winnipeg, Canada; 4 Département de pathologie et microbiologie, faculté de médecine vétérinaire, Université de Montréal, Saint-Hyacinthe, Canada; Naval Research Laboratory, UNITED STATES

## Abstract

An old world fruit bat *Pteropus giganteus*, held in captivity and suffering from necrosis of its wing digits, failed to respond to antibiotic therapy and succumbed to the infection. Samples submitted to the National Centre for Foreign Animal Disease were tested for viral infection. Vero E6 cells exhibited minor but unique cytopathic effects on second blind passage, and full CPE by passage four. Utilizing an unbiased random amplification technique from cell culture supernatant, we identified a bacterium belonging to the *Bradyrhizobiaceae*. Purification of cell culture supernatant on TY media revealed a slow growing bacterial isolate. In this study using electron microscopy, 16S rRNA gene analysis and whole genome sequencing, we identify a novel bacterial species associated with the site of infection belonging to the genus *Afipia*. This genus of bacteria is very diverse, with only a limited number of species characterized. *Afipia felis*, previously described as the etiological agent to cause cat scratch disease, and *Afipia septicemium*, most recently shown to cause disease in humans, highlight the potential for members of this genus to form a branch of opportunistic pathogens within the *Bradyrhizobiaceae*. Increased utilization of next generation sequencing and genomics will aid in classifying additional members of this intriguing bacterial genera.

## Introduction

*Pteropus giganteus*, more commonly referred to as an Indian flying fox, is a member of the *Pteropodidae* family. Indian flying foxes are found primarily within Asia, residing in tropical forests or swamps and serve as a reservoir for a number of viruses including Lyssavirus and henipaviruses [1,2]. Outbreaks of Hendra and Nipah viruses represent a serious public health threat. As a result, interest has increased significantly towards understanding flying foxes and their role in emerging infectious diseases.

Flying foxes are exotic animals occasionally kept in enclosures around the world. At one such enclosure, an Indian flying fox held in captivity was found to have progressive exudative skin lesions on a wing for a prolonged period of time, and necessitated two amputations prior to submission of the samples to the National Centre for Foreign Animal Disease (NCFAD) in Winnipeg. The skin formed small vesicles containing fluid which was released over time, leading to the skin sloughing away. Histologically, a neutrophilic vasculitis in the dermis with fibrinoid degeneration and necrosis was observed, with vesicles/bullae in the epidermis leading to sloughing of epidermis and fibrinosuppurative exudate forming on these ulcerated areas. An underlying viral infection or a "sterile" inflammatory/neutrophilic disorder was considered. Both topical and systemic antimicrobials were used to treat potential fungal and/or bacterial infections. The infected bat did not respond to antimicrobial therapy, resulting in two amputations to the affected wing and ultimately death, suggesting a systemic infection. Bat wing and vesicle samples were submitted to the NCFAD to exclude henipaviruses.

Bacterial pathogens afflicting bats have generally received much less attention. Currently very few bacterial species have been reported in flying foxes. Recently, it has been recognized that Leptospirosis, caused by the spirochete *Leptospira*, can be transmitted to rodents and potentially humans, through the urine of infected bats [3,4]. Leptospirosis is a biphasic disease, the first signs being flu like symptoms transitioning to a secondary phase which can lead to meningitis, liver damage and renal failure. Additional bacterial species reported in flying foxes include; *Listeria monocytogenes*, *Salmonella typhi*, *Salmonella typhimurium*, *Shigella flexneri* and *Shigella sonnei* [4]. Limited work has been performed to identify potential pathogens of bats or mutualistic bacteria found within their microbiome. Further understanding of this relationship will provide insight into potential risk factors associated with the natural flora of bats.

*Afipia* are gram negative alphaproteobacteria located within the family *Bradyrhizobiaceae*. The type species *Afipia felis*, was first described as the etiologic agent of cat scratch disease [5]. It was later determined to cause only a small number of cases while the majority of infections where due to *Bartonella henselae* [6]. Recent work has identified *Afipia* as *Legionella* like amoebae pathogens (LLAP) [7]. These bacteria have shown the ability to resist amoebae degradation and are considered to be a potential cause of unexplained pneumonia cases [8]. *A*. *felis* is capable of intracellular survival within macrophages by evading canonical endocytosis [9,10]. In light of these findings it has been suggested *Afipia* are potential human pathogens and this was recently confirmed by the identification of new *Afipia* species isolated from the blood of patients with unidentified infections [11,12].

In this report, using phylogenetic and next generation sequencing (NGS) supported by electron microscopy, we have identified a novel bacterium associated with the necrotic tissue of a flying fox as a member of the genus *Afipia*. Consequently, we have designated this bacterium *Afipia pteropus*.

## Materials and Methods

### Cell culture maintenance and growth conditions

African green monkey epithelial kidney cells (Vero E6) obtained from ATCC were maintained in Dulbecco Modified Eagle's medium (DMEM) supplemented with 10% fetal bovine serum (FBS) at 37°C with 5% CO_2_. Madin-Darby canine kidney (MDCK) cells were routinely maintained in Eagle's Minimum Essential medium (EMEM) supplemented with 10% FBS and incubated at 37°C with 5% CO_2_.

### Isolation Procedures

#### Virus isolation

Attempts for virus isolation were performed using Vero E6 cells (derived from the kidney cells of *Cercopithecus aethiops*) that were acquired from ATCC (Manassas, VA, U.S.A.). A 10% w/v homogenate of the bat wing digit was cultured on the VeroE6 cells. In addition, the vesicle or liquid sample from the bat abscess was passaged on Vero E6 grown at 37°C with 5% CO_2_ using DMEM media supplemented with 10% fetal bovine serum (Wisent. St. Jean Baptiste, QC, Canada).

Specifically, the VeroE6 cells were monitored for cytopathic effects (CPE) each day. Upon the first and second passage for each of the bat wing and vesicle samples, there was no apparent CPE observed. A third passage however, provided a unique CPE for each of the two bat wing samples. It was decided that a fourth passage be done to obtain a high titre of the unknown organism that was causing the CPE initially. As a result, after removal of cell debris using low speed centrifugation (ie. 3000 xg for 15 minutes), the fourth passage supernatant was frozen and used as stock for subsequent studies, such as electron microscopy and bacterial isolation.

#### Bacterial Isolation

To isolate bacteria, frozen cell culture supernatant from the forth passage on Vero E6 cells were streaked onto TY media and incubated at 30°C. Growth was closely monitored with single pure isolates cultivated and restreaked for cryopreservation. Bacterial cultures were maintained on TY medium containing 5% tryptone/peptone, 3% yeast extract, 1M calcium chloride media and 1.5% agar at 30°C.

### Electron Microscopy

Bacterial cell pellets or isolated bat vesicle and bat wing cell culture pellets were fixed in 2% paraformaldehyde, 2.5% glutaraldehyde in 0.1M Sorenson’s phosphate buffer pH 7.2, enrobed in 3% Agarose and post fixed in 1% osmium tetroxide. Samples were dehydrated through a graded series of ethanol and propylene oxide, followed by infiltration with a graded series of propylene oxide and araldite, embed 812 resin. Samples were then embedded in beem capsules and araldite, embed 812 resin, and capsules were cured at 68°C for 24 hours. 120nm sections were cut using a Leica Ultracut UCT, double stained with uranyl acetate and lead citrate. The specimen grids were examined in a Philips CM 120 transmission electron microscope operated at an accelerating voltage of 80kV, and at nominal instrument magnification of 10,000X. Digital images of the specimens were acquired by an AMT XR-611M CCD camera (AMT, Woburn, MA).

### Screening for unknown viruses

Nucleic acids (NA) were extracted from the bat wing and vesicle cell culture samples using the following protocol; 50 μl of cell culture supernatant was diluted in 200 μl of Dulbecco’s phosphate buffered saline (DPBS) (Sigma, St. Louis, MO, USA) and treated with 2 μl of Riboshredder (Epicentre, Madison, WI) and 2 ul of Omnicleave (Epicentre) at 37°C for 2 hours with 15 seconds of mixing at 1300 rpm every 3 minutes on ThermoMixer R (Eppendorf, Mississauga, ON, Canada). Total nucleic acid was then extracted from 200 μl of the nuclease treated samples using the QIAamp MinElute Virus Spin Kit (Qiagen, Toronto, ON, Canada). The isolated nucleic acids were assayed using the random amplification technique described by Nanda *et al* [13], as no prior knowledge is required of the genome or agent tested. Briefly, reverse transcriptase reactions were performed using the One-Step RT-PCR Kit (Qiagen) with the degenerate oligonucleotide primer (DOP) universal primers. The resulting amplicons were analyzed on a 1% agarose gel, purified with the MinElute Gel Extraction Kit (Qiagen) and cloned into pCR4-TOPO using the TOPO TA Cloning Kit for Sequencing (Invitrogen). Seventy-eight clones were sequenced using BigDye v3.1 and analyzed on a 3130XL Genetic Analyzer (Applied Biosystems). The sequences were then screened against the Nucleotide Collection (nr/nt) database via BLASTn (National Center for Biotechnology Information, Bethesda, MD) in order to determine the likely origin.

### 16S rRNA Analysis

Bacterial genomic DNA was isolated using the Qiagen Blood and Tissue Kit as recommended by manufacture. Amplification of the 16S rRNA gene was performed using the primers 27F - AGAGTTTGATCCTGGCTCAG and 1492R —GGTTACCTTGTTACGACTT [14]. The PCR protocol was as follows; an initial 5 min at 94°C, 94°C for 30 sec, 48°C for 30 sec, and 72°C for 2 min repeated for 29 cycles followed by a 10 min extension at 72°C. The resulting amplicon was gel extracted using the MinElute Gel Extraction Kit (Qiagen) and sequencing was performed as described above. Additional primers, located in S1 Table, were used to provide complete coverage of the resulting amplicon. Molecular Evolutionary Genetics Analysis version 5 (MEGA5) was used to align representative sequences via ClustalW and the phylogenetic relationship was assessed using maximum-likelihood [15].

### Genome sequencing and analysis

Genome sequencing of the bacterial isolate obtained from the Indian flying fox was performed as follows. A shotgun sequencing library was prepared using the Nextera XT DNA Sample Preparation Kit (Illumina) according to manufacturer’s instructions. Sequencing was performed on a MiSeq Benchtop Sequencer using the MiSeq Reagent Kit v2 (Illumina) generating 2 x 250 bp paired end reads. Sequence assembly was performed with the SPAdes genome assembler v. 2.5.0 [16] and annotations were added by the NCBI Prokaryotic Genome Annotation Pipeline Version 2.0 (http://www.ncbi.nlm.nih.gov/genome/annotation_prok/). This Whole Genome Shotgun project has been deposited at DDBJ/EMBL/GenBank under the accession AZSJ00000000. The version described in this paper is version AZSJ01000000.

An in house analysis pipeline was used to assign a taxonomic classification to the resulting coding sequences (CDS) based on the most recent common ancestor (MRCA). Briefly, each protein sequence was compared against the NCBI non-redundant (NR) database using BLASTp. Only matches with greater than 80% identity and a high-scoring segment pair (HSP) length greater than 80% of the query sequence length were considered. Those with bitscores in the top 1% were deemed to be equivalent hits and used for the MRCA assignment.

The predicted protein sequences were also analyzed against the MvirDB virulence database at Lawrence Livermore National Laboratory (LLNL) in a similar manner in order to assess the pathogenic potential of the isolate. BLASTp hits with a HSP length greater than 60% of the sequence length, 50% identity and an expect value greater than 0.0001 were considered significant.

### Ethics Statement

*Pteropus giganteus* field samples were obtained as a diagnostic submission. No live animal work was performed during this study.

## Results

### Isolation of potential virus from necrotic bat tissue

Failure of antibiotic treatment to alleviate the infection to the affected bat suggested a potential viral pathogen may be the cause of disease. Bat wing digit and vesicle samples submitted to the NCFAD were homogenized using a “MiniMix” blender and clarified by centrifugation. These homogenates were used for virus isolation, real time RT-PCR and conventional PCR detection. Because Indian flying foxes are reservoirs of henipaviruses, we wanted to ensure these viruses were not present. RNA was extracted from tissue homogenates using TriPure isolation reagent and a real time RT-PCR assay was performed as previously described [17,18]. Analysis of the data indicated no henipavirsues were present in either bat homogenate.

To further eliminate the possibility that a virus was responsible for the associated disease, virus isolation assays were performed using the Vero E6 cell line. Tissue and vesicle samples were used as an inoculum for confluent VeroE6 cells grown on T-25cm^2^ flasks. The flasks were monitored for the development of CPE. Upon two blind passages, no CPE was observed; however, upon passages three and four a non-lytic CPE was apparent. S1 Fig. diplayed control cells found in tight, healthy monolayers. However, in contrast, vesicle and bat wing treated Vero E6 cells showed cell clumping or aggregations of cells, indicative of a non-lytic CPE. This observation occurred after four days post infection (dpi), both in the third passage and even more so in the fourth passage of the unknown agent.

### Intracellular bacteria are present within mammalian cell culture

**Fig 1:**
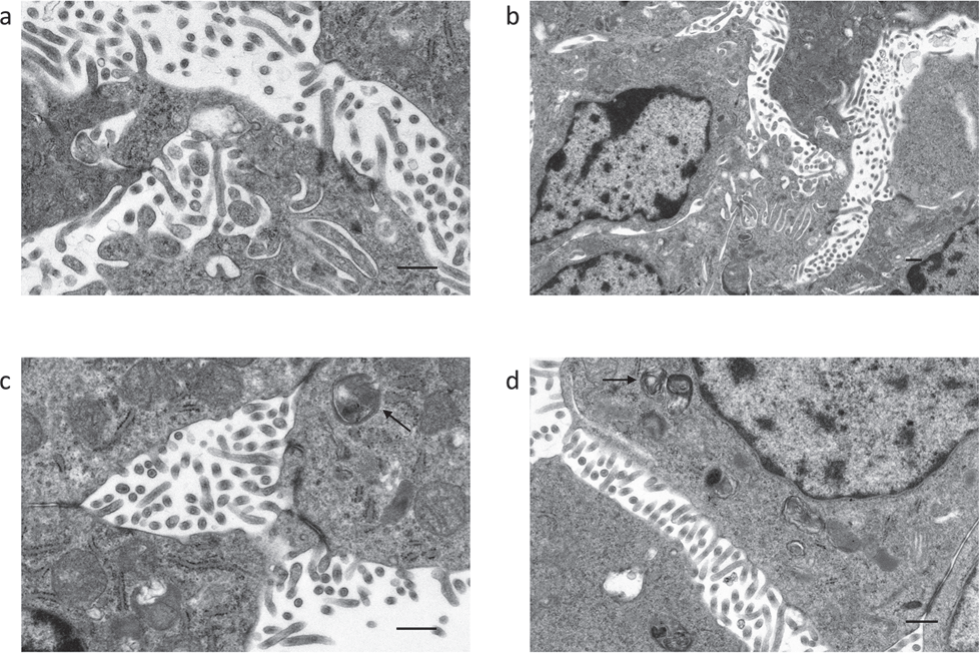
Representative electron microscopy images for cell culture. Vero E6 cells observed in; Panel (a) and (b) representing bat wing homogenate, (c) and (d) representing cell culture. Arrows indicate degraded *Afipia-*like cells. Scale bar = 2 μm.

The fourth passage Vero E6 cells inoculated with bat homogenate were examined by electron microscopy (EM) to determine if any source of infection could be detected. While no agents were detected in the cell culture supernatants by negative staining EM, cell samples embedded in epoxy resin and cut into thin sections for viewing revealed filamentous like projections at the surface extending into the extracellular space (Fig. 1A and Fig. 1B). These projections appear as cell derived appendages and not originating from a pathogenic agent. To ensure the filaments were not a member of the filoviridae, an RT-PCR assay to exclude filoviruses [19] confirmed no detectable virus was present in the samples. However, further analysis by EM identified *Afipia*-like structures located inside the Vero E6 cells. As shown in Fig. 1 C and Fig. 1D, intracellular *Afipia*-like structures are located in small clusters or localised individually.

### Identification of bacterial nucleic acid by unbiased random amplification

In coordination with electron microscopy analysis, we performed an unbiased random PCR assay with the potential for detecting unknown agents [13]. Isolated nucleic acid from cell culture bat wing and skin samples were amplified in a one step RT-PCR reaction and subsequently cloned into pCR4-TOPO. These fragments were sequenced on a 3130XL Genetic Analyzer and data was analysed by BLASTn. Interestingly, sequence analysis identified *Rhodopsuedomonas palustris* and *Bradyrhizobium japonicum*, both alpha proteobacteria, as the closest matches for the amplified nucleic acid.

### Bacterial isolation and 16S rRNA gene analysis

In an attempt to isolate the bacterium, cell culture aliquots were streaked onto TY agar medium. After 10 days, small colonies were observed correlating with the slow growth exhibited by many members of the *Bradyrhizobiaceae* (S2 Fig.). Colonies were greyish white, opaque, round and well defined. Single colonies were subsequently purified and frozen in 15% glycerol at -70°C.

**Fig 2:**
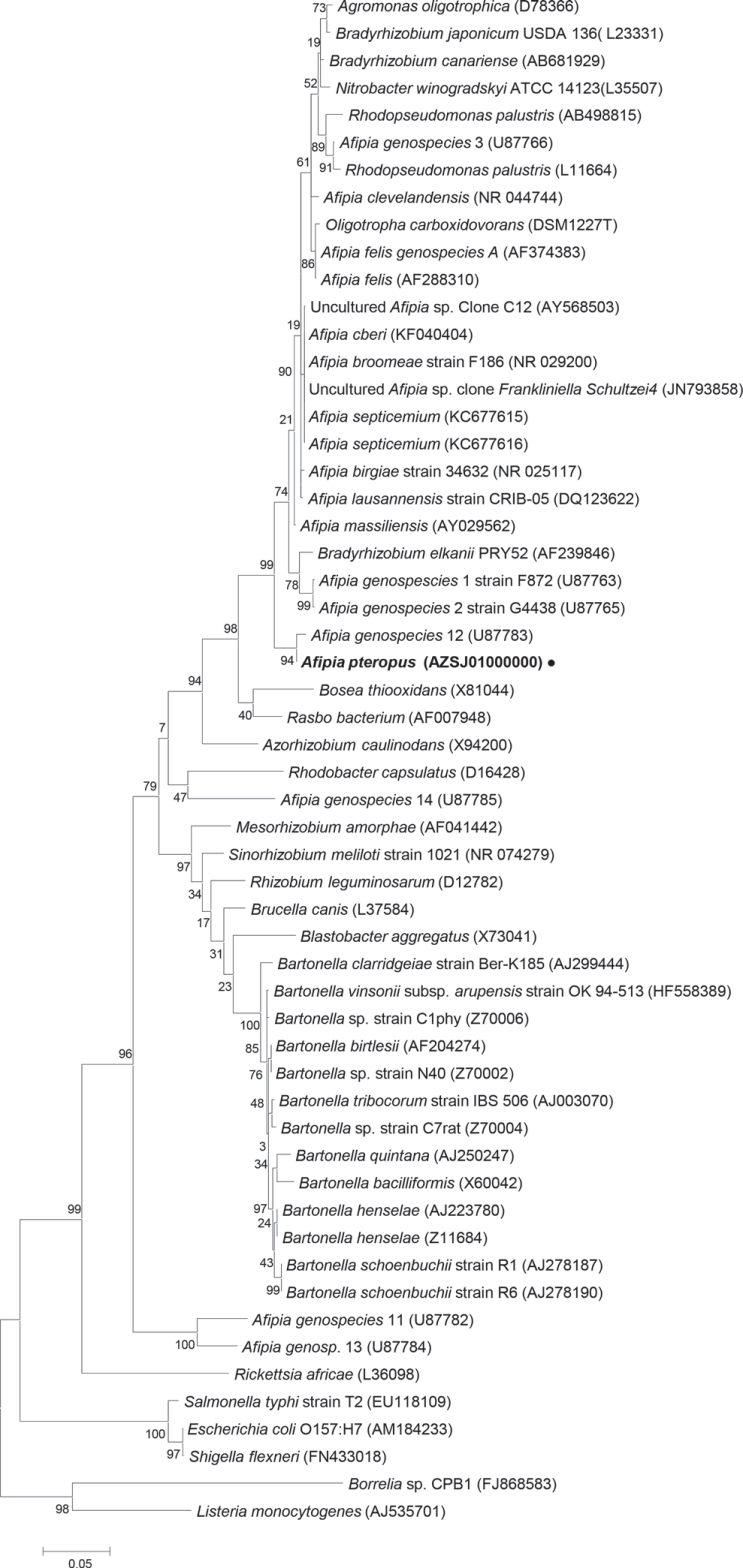
Phylogenetic analysis of the 16S rRNA gene. A maximum likelihood tree was constructed for the bat bacterial isolate using sequence obtained from NCBI BLASTn. Bacterial bat isolate is shown in bold as *Afipia pteropus* (AZSJ00000000). Sequences were obtained from both fully sequenced and partially sequenced genomes. Phylogenetic tree was constructed using the MEGA5 software[15].

To identify the bacteria, a 16S rRNA gene phylogenetic tree was generated. Total genomic DNA was prepared using a Qiagen Blood and Tissue Kit from bacterial colonies extracted from a TY plate. Following genomic DNA isolation, PCR amplification was performed using universal primers targeted to the 16S rRNA gene [20]. BLASTn analysis of the sequenced 16S rRNA gene generated a large number of matches to the bacterial family *Bradyrhizobiaceae*. A maximum likelihood tree was constructed using MEGA5 to ascertain the relationship between the unknown bacterium and other *Bradyrihzobium*. As indicated in Fig. 2, the bacterium isolated from the bat samples was most closely related to *Afipia genospecies* 12. Analysis of the phylogenetic tree indicates wide diversity of *Afipia* strains throughout. *Afipia* species are found in all branches aside from the γ-proteobacteria out-group as anticipated.

### Whole genome sequencing of bacterial isolate

**Fig 3:**
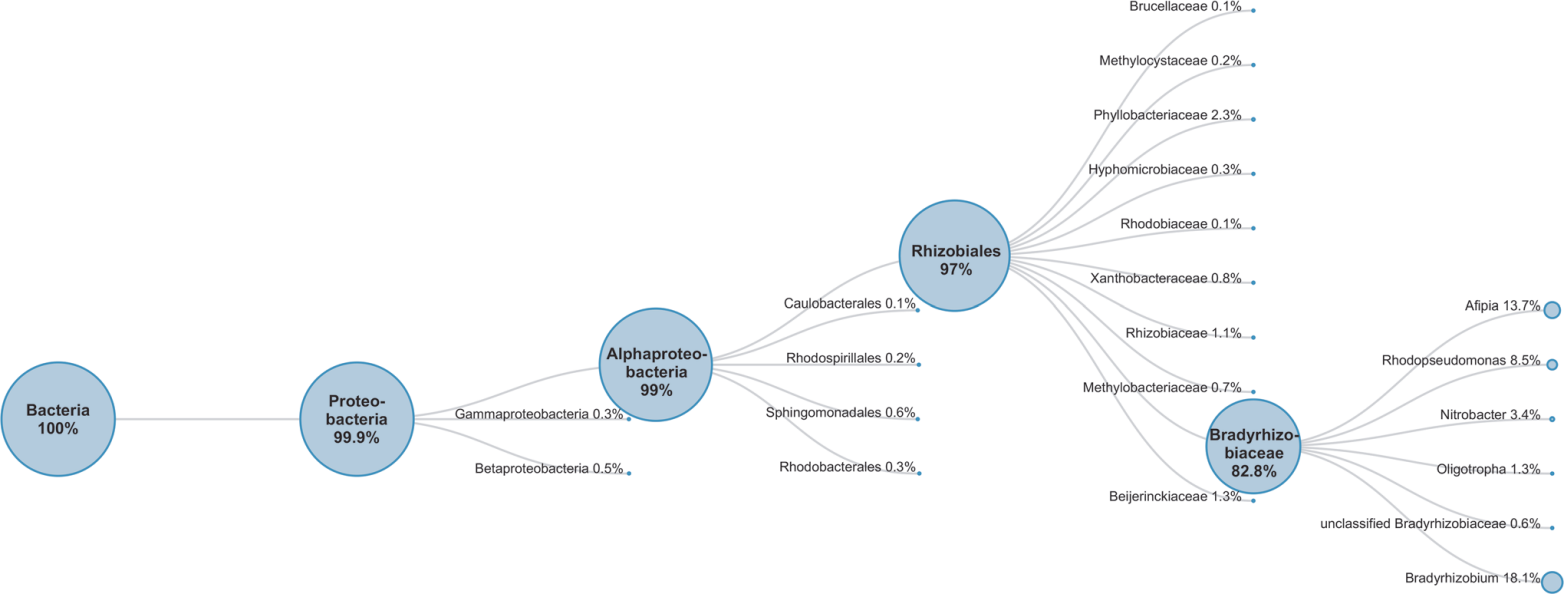
Lowest common ancestor (LCA) tree for genome sequence of bat bacterial isolate.

To characterize the bacterium further, a draft genome sequence was generated consisting of 7 contiguous sequences ranging in size from 1.9 kb to 2.9 Mbp with an average depth of coverage approaching 200x. The total combined length was 5,085,524 bp and harboured 4890 predicted genes, 4772 of which were protein coding and of those 1292 were deemed hypothetical proteins with no predicted function. A genome wide analysis of these genes using the MRCA approach (Fig. 3) clearly support the isolate as being a member of the *Bradyrhizobiaceae* family. However, when it came to classification down to the level of genus, 18.1% of the genes were most closely related to *Bradyrhizobium* and only 13.7% to Afipia. *Rhodopseudomonas* was the next most abundant at 8.5%. These results appear to be a slight contraction to those of the 16S rRNA gene analysis however it should be noted that *Bradyrhizobium* is more highly represented in the NR database than is *Afipia*. At the time of analysis the NR database contained approximately 6 times more unique gene entries for *Bradyrhizobium* spp than was found for *Afipia* spp (141,020 vs 21,931). Alternatively this could simply reflect the inter-relatedness among the *Bradyrhizobiaceae* family. This novel bacterium lies within the *Bradyrhizobiaceae*, and is expected to be a member of the genus *Afipia*. The genome sequence was deposited to DDBJ/EMBL/GenBank under the accession AZSJ00000000.

Analysis against the MvirDB virulence database (S2 Table) identified 18 genes associated with antibiotic resistance and 42 with pathogenicity islands. Ten genes were identified as transcription factors, 68 as general virulence proteins, and 1 as a protein toxin. Putative antibiotic resistance based on predicted gene function includes; neomycin, kanamycin, chloramphenicol, tetracycline, fosmidomycin and beta-lactams. In addition, the isolate also encodes for capsular polysaccharide synthesis, lipopolysaccharide and transport system virulence genes.

### Electron microscopy of bacterial isolate

**Fig 4:**
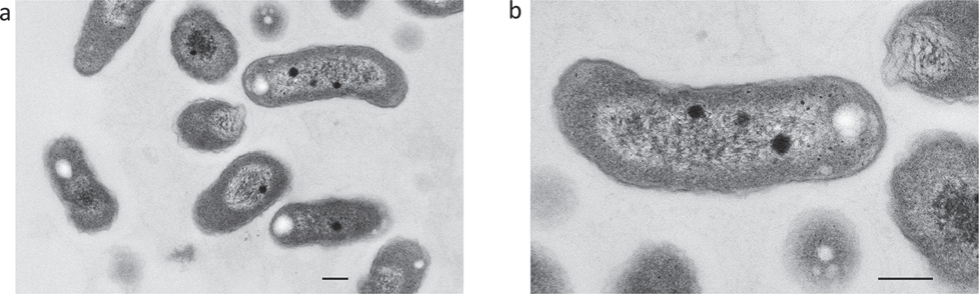
Thin section photomicrograph images of *Afipia pteropus*. Bacterial cells were grown on TY agar plates at 30°C for 10 days. Colonies were pooled and placed in fixative for processing. Gram negative cell wall structure is easily discernible by dark outline. Fibrous material inside the cell resembles large amounts of nucleic acid. A) 13000X magnification and B) 28000X magnification. Scale bar represents 200nm.

The bacterial colonies isolated on TY plates were pelleted and processed for electron microscopy. Thin sections were analyzed to determine ultrastructure of the new bacterial isolate. Bacterial cells on average are approximately 1 micron in length and 0.4 microns in diameter (Fig. 4). They are rod shaped with characteristic gram negative cell wall structure. The majority have some degree of fibrous like material centrally located and electron dense bodies typical to electron micrographs. In general, the overall cell structure and shape matches well with other members of the *Afipia* species [10,12,21,22].

## Discussion

Bats are unique mammals which have recently gained increased attention due to their association with emerging highly pathogenic viruses. While they serve as reservoirs for a number of serious human pathogens such as; Hendra and Nipah viruses, SARS coronavirus, or Ebola/Marburg viruses, very little research has been performed to characterize their microflora. This study identified a new bacterial species associated with infection of an Indian flying fox. We have identified the bacterial isolate as a unique *Afipia* species, most closely related by 16S rRNA gene analysis to *Afipia genospecies* 12. We are tentatively annotating this new bacterial isolate as *Afipia pteropus*. Very little is known about this genus with the most recognised type strain being *Afipia felis*, originally thought to cause cat scratch disease (CSD) [6,23]. Recent work by Lo *et al*. documented the discovery of *A*. *septicemia* which produced a virus like disease in human patients [11,12]. It is possible that the newly identified *Afipia pteropus* may have had a similar course of infection in the affected bat, although the only apparent clinical sign was progressive necrosis of the wing digits in the early stages of the disease.

In general, the cell morphology of the *Afipia* genus is a slightly bent rod shaped bacterium [10]. Appropriately, *A*. *pteropus* closely resembles other members of the *Afipia*, including the recently identified *A*. *septicemia*, by analysis using electron microscopy. Interestingly, *Afipia* species are capable of intracellular growth, both inside amoeba, macrophages or other mammalian cell lines [9,10,21,22,24]. It has been previously shown *A*. *felis* are capable of inducing macropinocytosis to gain entry into macrophages similar to other intracellular pathogens [9,25]. In contrast, non-professional phagocytes infected with *A*. *felis* undergo classical endocytic pathway dynamics [22]. Although we do not know the mechanism of internalization, we were able to detect *Afipia*-like particles within Vero E6 cells. This internalisation may be important to pathogenesis of this bacterial species. It has been previously documented using microscopy and ultrastructure analysis, that *A*. *felis* induce cells to produce filamentous fibriles [22]. In this study, Vero E6 cells similarly displayed long filamentous appendages within the extracellular space.

The *Bradyrhizobiaceae* are an extremely diverse group of bacteria and it has been suggested this group be divided to more accurately represent the bacteria within it [23]. In general, the *Bradyrhizobiaceae* are defined as bacteria found within soil or water, either as saprophytic or symbiotic organisms. Based on our 16S rRNA analysis, the *Afipia* are located within the *Bradyrhizobiaceae*, however *Afipia genospecies* 14 is located next to *Rhdobacter capsulatus* of the *Rhodobacteraceae*. Further, *A*. *genospecies* 11 and 13 are more distantly related than *Bartonella* species further exemplifying the diversity of this genus. Although the majority of *Afipia* are more closely related, the boot strap values at nodes separating the various species appear low. Classification of a new *Afipia* family may alleviate the skewed boot strap values by partitioning out the *Bradyrhizobium* species. Future placement of newly sequenced *Afipia* isolates will provide greater depth to understanding this genus. From our phylogenetic tree we can infer that most, if not all the bacteria are capable of infection whether it be a symbiont or a pathogen.

The overall size of *A*. *pteropus* genome is close to 5 Mb, similar to the fully sequenced *Rhodopseudomonas palustris*, yet smaller than other sequenced *Bradyrhizobiacae*. The genome sequence predicts a metabolically diverse organism, with suggested motility based on the presence of genes encoding a flagellar apparatus. Interestingly, similar to *Agrobacterium tumefaciens*, a type IV virulence secretion system was predicted by genome sequence. These systems are used for transfer of proteins or protein-nucleic acid complexes out of the bacterial cell into a prokaryotic or Eukaryotic host [26]. In addition, proteins predicted for transport through a type VII secretion system are encoded by the genome. This system is most recently known for its role in *Mycobacterium* extracellular protein transport [27,28]. If functional, both of these secretion systems could play a role in pathogenesis. The genome sequence further predicts a large number of antibiotic resistance and virulence genes by analysis through the MvirDB virulence database. The high abundance of antibiotic resistance genes may account for the ineffectiveness of drugs used during treatment. Genome sequence analysis identified many putative antibiotic resistance genes including those for beta-lactams, fluoroquinolones, kanamycin and neomycin among others. The course of antibiotic administration during treatment included among others BNP topical ointment and amphotericin B. Systemic antibiotics were also administered including amoxicillin, enrofloxacin, TMS and itraconazole. There appears to be a direct correlation between drug treatment and antibiotic resistance likely explaining the failed course of treatment. In addition, extracellular capsular polysaccharide was identified to be closely related to *Staphlococcus aureus*. Capsules enhance the virulence of bacterial strains by resisting phagocytosis and prolonging survival. Extracellular capsules further contribute to biofilm formation. Biofilms are composed of an extracellular matrix surrounding bacteria forming surface associated communities. They provide shared resources for survival and help resist outside pressures such as antibiotic therapy [29,30]. Together these factors may allow for long term chronic infections.

Although work has progressed toward understanding *Afipia*, it is unclear as to what niche these bacteria inhabit. Previous work has detected *Afipia* spp. in hospital and drinking water. Moreover, it was shown *Afipia* were able to infect amoebae [24,31,32]. These data suggest *Afipia* may generally survive in water networks as free living organisms. In light of this, case files from acquired human infections suggest *Afipia* may also be an opportunistic human pathogen [23,24,32]. The MvirDB database for *A*. *pteropus* identified many genes that may contribute to pathogenesis. Further, like *A*. *felis*, intracellular entry is coordinated in a similar fashion to *Legionella* [9,10,22]. Unidentified infections can often mimic many agents as outlined by Lo *et al*. making treatment very difficult [11,12]. The expanded use of NGS will continue to aid in the identification and treatment of future infections ultimately providing better surveillance and care.

## Supporting Information

S1 Fig Vero E6 cell culture after four passages infected with bat wing homogenate. A) Uninfected control cells showing regular growth, B) Cells infected with bat wing homogenate showing mild CPE.
(TIF)

S2 Fig Colony morphology of *A*. *pteropus* by phase contrast microscopy. Pure bacterial cultures were grown on TY agar petri plates at 30°C for 10 days. Images were obtained using an Olympus CKX41 inverted microscope equipped with a DP71 Olympus camera.
(TIF)

S1 Table Primers for 16S rRNA gene sequencing (PDF)

S2 Table Predicted Protein sequences were assessed against the MvirDB virulence database (PDF)
