# Supplementary figures and images for: Identification of a Novel Afipia Species Isolated from an Indian Flying Fox
Source: PLoS One. 2015 Apr 15;10(4):e0121274. doi: 10.1371/journal.pone.0121274 (PMC4398416; doi:10.1371/journal.pone.0121274)

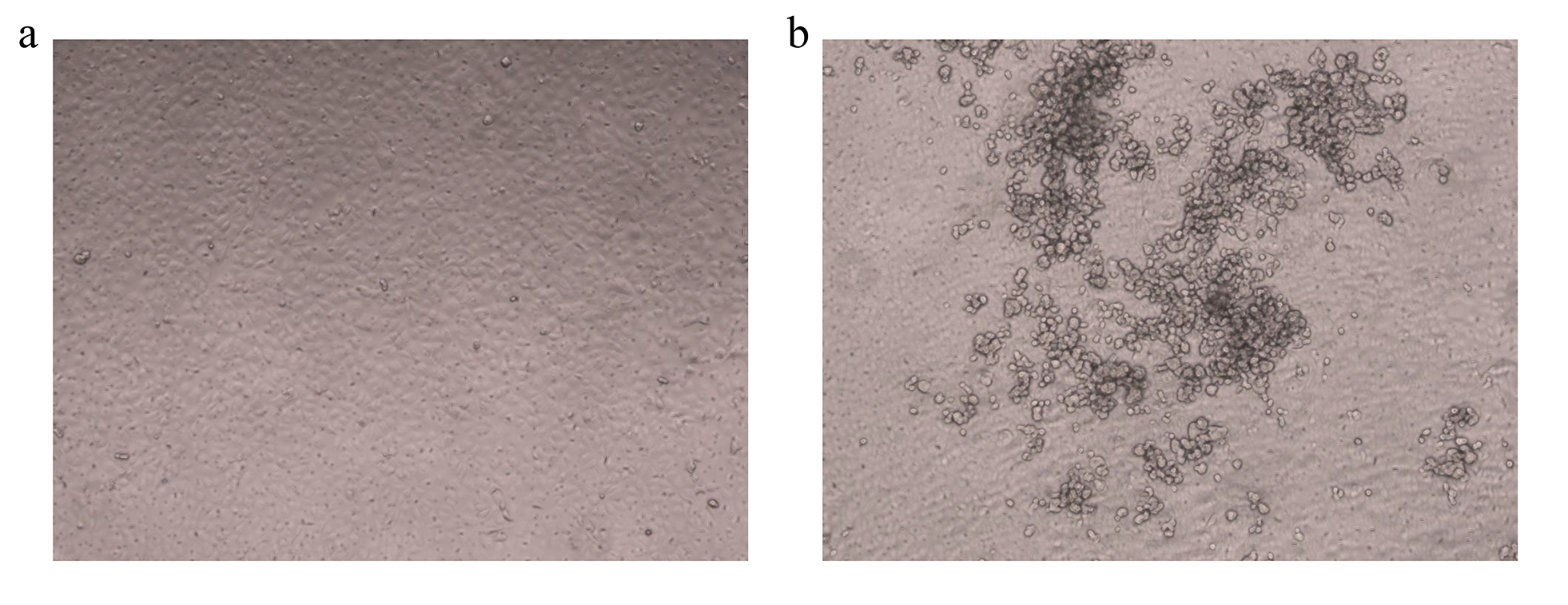

Supplement: S1 Fig — A) Uninfected control cells showing regular growth, B) Cells infected with bat wing homogenate showing mild CPE. (TIF) [file pone.0121274.s001.tif]

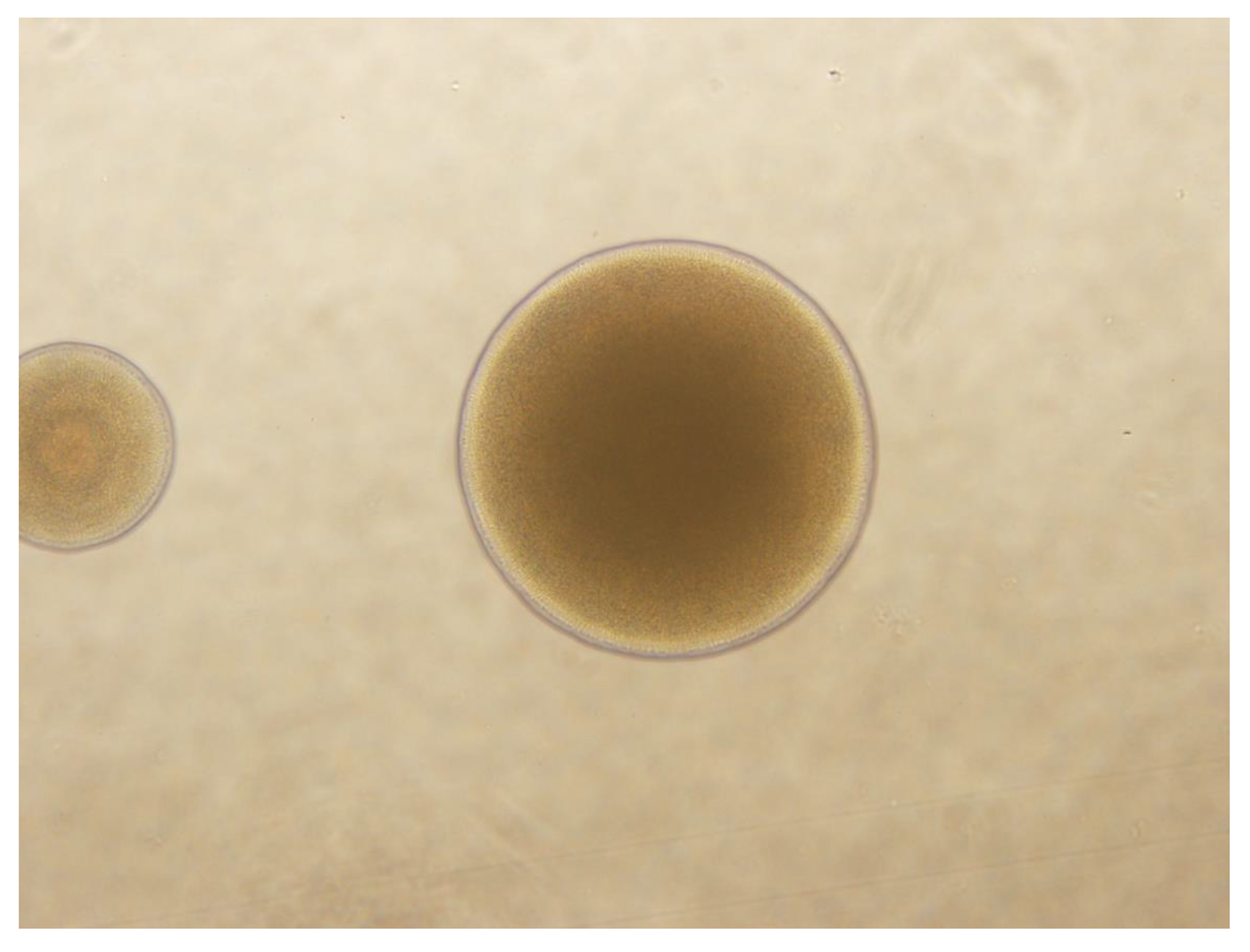

Supplement: S2 Fig — Pure bacterial cultures were grown on TY agar petri plates at 30°C for 10 days. Images were obtained using an Olympus CKX41 inverted microscope equipped with a DP71 Olympus camera. (TIF) [file pone.0121274.s002.tif]
