# Supplementary material for: Identification of a Novel Afipia Species Isolated from an Indian Flying Fox
Source: PLoS One. 2015 Apr 15;10(4):e0121274. doi: 10.1371/journal.pone.0121274 (PMC4398416; doi:10.1371/journal.pone.0121274)
Supplement: S1 Table — (PDF) [file pone.0121274.s003.pdf]

Supplemental Table 1

Primers for 16S rRNA gene sequencing

Primer name	5'-3'
BSP8	CGGAATCACTGGGCGTAAA
BSP9	CGCTTTACGCCCAGTGATT
BSP10	CCGTCCTTAGTTGCTACCATTC
BSP11	GTCTCCTTAGAGTGCTCAACTG
