# Supplementary material for: Identification of a Novel Afipia Species Isolated from an Indian Flying Fox
Source: PLoS One. 2015 Apr 15;10(4):e0121274. doi: 10.1371/journal.pone.0121274 (PMC4398416; doi:10.1371/journal.pone.0121274)
Supplement: S2 Table — (PDF) [file pone.0121274.s004.pdf]

Supplemental Table 2

Predicted Protein sequences were assessed against the MvirDB virulence database

Read Name	Bitscore	Expect	HSP Length	Percent Match	Virulence Factor ID	Virulence Factor Type	Short Description
X566_02865	1051	0	1025	63.8	3300	antibiotic resistance	putative AcrB/AcrD/AcrF family membrane protein
X566_02930	433	0	469	50.75	3452	antibiotic resistance	B protein, Tri12 like (fungal trichothecene)
X566_03220	1370	0	1024	68.95	15209	antibiotic resistance	SubName: Full=Hydrophobe/amphiphile efflux-1 family protein;
X566_03225	296	0	325	52.31	15208	antibiotic resistance	SubName: Full=HlyD family secretion protein;
X566_04760	383	0	382	57.33	24627	antibiotic resistance	fosmidomycin resistance protein [Yersinia pseudotuberculosis IP 31758]
X566_06545	299	0	292	52.05	3290	antibiotic resistance	putative metallo-beta-lactamase family protein
X566_06580	203	0	255	50.59	3480	antibiotic resistance	Aminoglycoside phosphotransferase
X566_13650	901	0	1052	50.57	24240	antibiotic resistance	multidrug efflux RND transporter MexF [Pseudomonas syringae pv. tomato str. DC3000]
X566_16675	1034	0	1009	54.91	24212	antibiotic resistance	hydrophobe/amphiphile efflux-1 HAE1 [Pseudomonas fluorescens Pf0-1]
X566_16740	294	0	331	51.66	22911	antibiotic resistance	efflux transporter, RND family, MFP subunit [Burkholderia cenocepacia MC0-3]
X566_16745	1344	0	1043	73.25	22949	antibiotic resistance	hydrophobe/amphiphile efflux-1 (HAE1) family protein [Burkholderia multivorans ATCC 17616]
X566_17375	1229	0	1028	63.62	15209	antibiotic resistance	SubName: Full=Hydrophobe/amphiphile efflux-1 family protein;
X566_18880	519	0	394	72.08	3508	antibiotic resistance	tetracycline-efflux transporter
X566_19355	102	2.00E-028	107	56.07	23347	antibiotic resistance	multidrug efflux protein [Escherichia coli E24377A]
X566_19785	310	0	294	50.68	3290	antibiotic resistance	putative metallo-beta-lactamase family protein
X566_19805	393	0	370	51.62	169	antibiotic resistance	putative aspartate amino transferase [Kluyvera ascorbata]
X566_22830	283	0	242	52.07	22454	antibiotic resistance	RecName: Full=Beta-lactamase OXA-9; AltName: Full=Penicillinase; AltName: Full=Oxacillinase; Flags: Precursor
X566_24230	475	0	547	62.52	24656	antibiotic resistance	putative cation:proton antiport protein [Yersinia enterocolitica subsp. enterocolitica 8081]

X566_00225	393	0	413	52.78	19085	pathogenicity island	secreted protein [Streptomyces coelicolor A3(2)]
X566_02220	404	0	320	63.75	15643	pathogenicity island	hypothetical protein blr1588 [Bradyrhizobium japonicum USDA 110]
X566_02270	191	0	184	51.09	15493	pathogenicity island	hypothetical protein blr8041 [Bradyrhizobium japonicum USDA 110]
X566_03120	281	0	297	50.17	18830	pathogenicity island	oligopeptide ABC transporter, ATP-binding protein [Streptococcus agalactiae NEM316]
X566_03915	293	0	254	52.36	18831	pathogenicity island	oligopeptide ABC transporter, ATP-binding protein [Streptococcus agalactiae NEM316]
X566_03920	316	0	268	59.33	19440	pathogenicity island	putative peptide transport system ATP-binding ABC transporter protein [Sinorhizobium meliloti 1021]
X566_04865	343	0	347	53.03	20941	pathogenicity island	glucose dehydrogenase B [Xylella fastidiosa 9a5c]
X566_05630	72	1.00E-017	52	59.62	16203	pathogenicity island	cold shock-like protein cspB [Escherichia coli CFT073]
X566_05745	83	6.00E-022	60	68.33	15506	pathogenicity island	hypothetical protein bsr8028 [Bradyrhizobium japonicum USDA 110]
X566_06135	299	0	290	55.52	18096	pathogenicity island	hypothetical protein mll5466 [Mesorhizobium loti MAFF303099]
X566_06495	649	0	502	60.16	20445	pathogenicity island	4-hydroxyphenylacetate catabolism [Salmonella enterica subsp. enterica serovar Typhimurium str. LT2]
X566_06885	91	1.00E-024	72	52.78	20588	pathogenicity island	translation initiation factor IF-1 [Salmonella enterica subsp. enterica serovar Typhimurium str. LT2]
X566_06930	141	4.06E-044	99	73.74	17305	pathogenicity island	urease subunit gamma [Escherichia coli O157:H7 str. Sakai]
X566_06940	154	0	101	72.28	17304	pathogenicity island	urease subunit beta [Escherichia coli O157:H7 str. Sakai]
X566_07665	239	0	205	53.17	20545	pathogenicity island	putative metallo-beta-lactamase [Salmonella enterica subsp. enterica serovar Typhimurium str. LT2]
X566_09265	92	2.00E-025	71	60.56	15610	pathogenicity island	hypothetical protein bsr1621 [Bradyrhizobium japonicum USDA 110]
X566_09270	387	0	402	57.71	15611	pathogenicity island	conjugal transfer protein [Bradyrhizobium japonicum USDA 110]
X566_09275	308	0	306	57.19	15612	pathogenicity island	conjugal transfer protein [Bradyrhizobium japonicum USDA 110]

X566_09280	366	0	227	74.01	15613	pathogenicity island	conjugal transfer protein TrbF [Bradyrhizobium japonicum USDA 110]
X566_09480	251	0	210	58.1	16488	pathogenicity island	hypothetical protein c1285 [Escherichia coli CFT073]
X566_09500	457	0	287	77.35	15619	pathogenicity island	antirestriction protein [Bradyrhizobium japonicum USDA 110]
X566_10100	237	0	171	61.99	20476	pathogenicity island	3-hydroxydecanoyl-(acyl carrier protein) dehydratase [Salmonella enterica subsp. enterica serovar Typhimurium str. LT2]
X566_11245	86	1.00E-022	73	52.05	15511	pathogenicity island	hypothetical protein bsl8023 [Bradyrhizobium japonicum USDA 110]
X566_12695	69	2.00E-016	66	50	16196	pathogenicity island	cold shock-like protein cspI [Escherichia coli CFT073]
X566_13605	322	0	315	55.24	16120	pathogenicity island	ABC-type transporter, ATPase component [Corynebacterium glutamicum ATCC 13032]
X566_13965	317	0	318	54.09	20583	pathogenicity island	thioredoxin reductase [Salmonella enterica subsp. enterica serovar Typhimurium str. LT2]
X566_15825	483	0	515	50.49	19089	pathogenicity island	methylmalonyll-CoA mutase [Streptomyces coelicolor A3(2)]
X566_16160	317	0	288	53.47	19440	pathogenicity island	putative peptide transport system ATP-binding ABC transporter protein [Sinorhizobium meliloti 1021]
X566_16165	282	0	268	56.72	19440	pathogenicity island	putative peptide transport system ATP-binding ABC transporter protein [Sinorhizobium meliloti 1021]
X566_16640	266	0	276	52.17	19440	pathogenicity island	putative peptide transport system ATP-binding ABC transporter protein [Sinorhizobium meliloti 1021]
X566_17760	128	5.00E-037	102	55.88	15471	pathogenicity island	hypothetical protein blr8063 [Bradyrhizobium japonicum USDA 110]
X566_18105	272	0	262	55.73	19440	pathogenicity island	putative peptide transport system ATP-binding ABC transporter protein [Sinorhizobium meliloti 1021]
X566_18110	297	0	314	52.87	19440	pathogenicity island	putative peptide transport system ATP-binding ABC transporter protein [Sinorhizobium meliloti 1021]
X566_18615	371	0	336	60.12	18220	pathogenicity island	alcohol dehydrogenase, zinc-containing [Pseudomonas putida KT2440]
X566_19680	280	0	250	55.6	18820	pathogenicity island	hypothetical protein gbs0137 [Streptococcus agalactiae NEM316]

X566_19895	69	1.00E-016	50	56	16196	pathogenicity island	cold shock-like protein cspI [Escherichia coli CFT073]
X566_20380	227	0	246	51.22	18814	pathogenicity island	hypothetical protein gbs0131 [Streptococcus agalactiae NEM316]
X566_20860	899	0	608	73.68	15599	pathogenicity island	glucosamine--fructose-6-phosphate aminotransferase [Bradyrhizobium japonicum USDA 110]
X566_21780	139	3.00E-042	128	52.34	18893	pathogenicity island	30S ribosomal protein S9 [Streptococcus agalactiae NEM316]
X566_21785	152	0	144	51.39	18892	pathogenicity island	50S ribosomal protein L13 [Streptococcus agalactiae NEM316]
X566_23080	1051	0	1069	50.8	18840	pathogenicity island	DNA-directed RNA polymerase subunit beta` [Streptococcus agalactiae NEM316]
X566_23345	167	0	145	58.62	18221	pathogenicity island	isoquinoline 1-oxidoreductase, alpha subunit, putative [Pseudomonas putida KT2440]
X566_03520	181	0	208	56.73	3399	protein toxin	hemolysin III
X566_02765	413	0	405	53.33	11888	transcription factor	SubName: Full=Probable two-component response regulator;
X566_04600	431	0	275	76.36	15217	transcription factor	RecName: Full=RNA polymerase sigma factor;
X566_06990	209	0	167	57.49	15218	transcription factor	RecName: Full=RNA polymerase sigma factor;
X566_07650	193	0	158	70.25	10963	transcription factor	SubName: Full=Response regulator;
X566_08525	150	0	102	74.51	10833	transcription factor	RecName: Full=Integration host factor subunit beta; Short=IHF-beta;
X566_14205	592	0	450	66	10283	transcription factor	RecName: Full=Transcriptional regulatory protein flbD;
X566_14740	234	0	171	68.42	10855	transcription factor	SubName: Full=Response regulator;
X566_17290	83	7.00E-020	56	64.29	10709	transcription factor	SubName: Full=FlbD;
X566_20540	821	0	480	93.54	12011	transcription factor	RecName: Full=Nitrogen assimilation regulatory protein;
X566_21680	71	1.00E-016	65	50.77	2800	transcription factor	dna binding
X566_00170	145	4.20E-045	110	60	14023	virulence protein	ORF50 protein of pB171 [Escherichia coli]
X566_02355	205	0	217	54.84	7262	virulence protein	enterochelin transporter (uptake) [Shigella flexneri 2a str. 2457T]
X566_02360	137	3.00E-041	132	56.06	7263	virulence protein	energy transduction protein [Salmonella typhimurium LT2]
X566_02990	456	0	472	50.85	3230	virulence protein	putative GDP-mannose pyrophosphorylase
X566_04915	250	0	281	53.38	13862	virulence protein	putative ABC transporter membrane protein [Escherichia coli]
X566_04935	322	0	199	75.88	3408	virulence protein	Fe dependent superoxide dismutase
X566_05120	902	0	851	57.34	15128	virulence protein	SubName: Full=ClpB protein;
X566_06830	425	0	420	50.95	3320	virulence protein	pilus assembly, secretory protein kinase

X566_06945	914	0	569	79.26	10906	virulence protein	RecName: Full=Urease subunit alpha; EC=3.5.1.5; AltName: Full=Urea amidohydrolase subunit alpha;
X566_07235	204	0	215	53.49	7256	virulence protein	ExbB [Escherichia coli O157:H7 str. Sakai]
X566_07240	157	0	143	58.04	7261	virulence protein	enterochelin transporter (uptake) [Shigella flexneri 2a str. 2457T]
X566_07360	1234	0	947	65.79	3425	virulence protein	
X566_07430	1254	0	755	84.5	12371	virulence protein	SubName: Full=EnzymeI-Ntr;
X566_08220	261	0	292	52.74	26987	virulence protein	adhesin protein AP33-2 [Trichomonas vaginalis]
X566_08490	117	1.00E-034	93	50.54	15145	virulence protein	SubName: Full=Thioredoxin;
X566_08610	486	0	526	51.33	15220	virulence protein	SubName: Full=RNA polymerase sigma-54 factor rpon;
X566_08795	786	0	597	66	26468	virulence protein	organellar heat shock protein [Eimeria tenella]
X566_09985	259	0	254	50.79	13923	virulence protein	hypothetical protein [Escherichia coli]
X566_09990	165	0	193	55.96	13924	virulence protein	hypothetical protein [Escherichia coli]
X566_10465	705	0	475	70.32	3404	virulence protein	catalase
X566_10790	1053	0	1045	57.99	15210	virulence protein	SubName: Full=Hydrophobe/amphiphile efflux-1 family protein;
X566_11625	432	0	453	50.33	15206	virulence protein	SubName: Full=UDP-glucose 6-dehydrogenase 2; EC=1.1.1.22;
X566_12440	331	0	343	59.77	3466	virulence protein	motor torque providing
X566_12475	339	0	330	55.15	26401	virulence protein	fructose-1,6-bisphosphate aldolase [Toxoplasma gondii]
X566_12640	182	0	134	65.67	30491	virulence protein	RecName: Full=Outer membrane lipoprotein omp16; AltName: Full=16.5 kDa minor OMP; Short=16 kDa OMP; AltName: Full=Minor outer membrane protein omp16; Flags: Precursor;
X566_13210	624	0	526	67.3	8350	virulence protein	chaperonin GroEL [Legionella pneumophila subsp. pneumophila str. Philadelphia 1]
X566_13880	222	0	240	50	15195	virulence protein	SubName: Full=Putrescine transport system permease protein;
X566_13910	348	0	315	55.56	3387	virulence protein	iron(III) binding periplasmic protein precursor
X566_13990	1271	0	1120	58.84	15125	virulence protein	SubName: Full=Carbamoyl-phosphate synthase large chain; EC=6.3.5.5;
X566_14015	392	0	381	50.39	15124	virulence protein	SubName: Full=Carbamoyl-phosphate synthase small chain; EC=6.3.5.5;

X566_14210	124	6.00E-037	91	67.03	12330	virulence protein	RecName: Full=Flagellar motor switch protein FliN;
X566_15030	158	0	156	50	7196	virulence protein	bacterioferritin, iron storage and detoxification protein [Escherichia coli K12]
X566_15390	56	2E-10	42	50	31168	virulence protein	RecName: Full=ATP-dependent Clp protease ATP-binding subunit ClpX;
X566_16930	129	1.00E-038	115	55.65	2489	virulence protein	aspartate alpha-decarboxylase [Mycobacterium tuberculosis H37Rv]
X566_17460	435	0	332	62.35	12323	virulence protein	SubName: Full=WbnF;
X566_17465	351	0	285	61.75	3244	virulence protein	glucose-1-phosphate thymidylyltransferase
X566_17475	366	0	349	55.87	3245	virulence protein	dTDP-glucose 4,6-dehydratase
X566_17480	160	0	148	53.38	3243	virulence protein	dTDP-6-deoxy-D-glucose-3,5 epimerase
X566_17500	517	0	364	67.86	7853	virulence protein	lipopolysaccharide biosynthesis protein [Bordetella pertussis Tohama I]
X566_17505	250	0	186	65.59	7852	virulence protein	acetyltransferase [Bordetella pertussis Tohama I]
X566_17510	553	0	346	73.41	7851	virulence protein	oxidoreductase [Bordetella pertussis Tohama I]
X566_17540	400	0	365	52.6	2709	virulence protein	capsular polysaccharide synthesis enzyme Cap8G [Staphylococcus aureus subsp. aureus MW2]
X566_17550	447	0	332	63.55	2707	virulence protein	capsular polysaccharide synthesis enzyme Cap8E [Staphylococcus aureus subsp. aureus MW2]
X566_17570	376	0	361	55.4	7854	virulence protein	UDP-N-acetylglucosamine 2-epimerase [Bordetella pertussis Tohama I]
X566_20405	887	0	1015	51.92	12272	virulence protein	RecName: Full=Multidrug resistance protein mdtB; AltName: Full=Multidrug transporter mdtB;
X566_20560	81	1.00E-020	73	50.68	15136	virulence protein	RecName: Full=Protein hfq;
X566_20625	308	0	262	59.54	3471	virulence protein	Dnase
X566_21065	147	1.96E-044	110	64.55	13464	virulence protein	single-strand DNA-binding protein [Salmonella typhimurium LT2]
X566_21190	288	0	301	55.15	11711	virulence protein	SubName: Full=Probable protein-export membrane protein;
X566_21195	558	0	537	56.98	11711	virulence protein	SubName: Full=Probable protein-export membrane protein;
X566_21240	245	0	253	59.29	3469	virulence protein	sec-independent protein translocase
X566_21250	64	8E-015	66	59.09	3470	virulence protein	sec-independent protein translocase
X566_21280	336	0	393	56.23	15223	virulence protein	RecName: Full=Deoxyguanosinetriphosphate triphosphohydrolase-like protein;
X566_21710	534	0	381	67.98	31168	virulence protein	RecName: Full=ATP-dependent Clp protease ATP-binding subunit ClpX;

X566_21715	214	0	190	56.32	8175	virulence protein	ATP-dependent Clp protease proteolytic subunit [Listeria monocytogenes EGD-e]
X566_21750	472	0	472	51.91	13817	virulence protein	glutamine synthetase GLNA1 (glutamine synthase) (GS-I) [Mycobacterium tuberculosis H37Rv]
X566_22035	925	0	752	63.3	15221	virulence protein	RecName: Full=GTP pyrophosphokinase rsh; EC=2.7.6.5; AltName: Full=ATP:GTP 3`-pyrophosphotransferase; AltName: Full=(p)ppGpp synthetase;
X566_22065	89	8.00E-023	139	51.8	12297	virulence protein	RecName: Full=Large-conductance mechanosensitive channel;
X566_22935	646	0	444	73.2	3409	virulence protein	
X566_23125	70	6.00E-017	59	62.71	3410	virulence protein	
X566_23320	520	0	447	56.82	2755	virulence protein	glutamate dehydrogenase
X566_23895	625	0	526	65.59	8350	virulence protein	chaperonin GroEL [Legionella pneumophila subsp. pneumophila str. Philadelphia 1]
X566_24450	83	1.00E-021	86	59.3	11615	virulence protein	RecName: Full=Flagellar biosynthetic protein FliQ;
X566_24460	150	0	136	54.41	3426	virulence protein	flagellar basal body rod protein
X566_24475	255	0	223	65.02	11844	virulence protein	RecName: Full=Flagellar biosynthetic protein fliP;
X566_24555	406	0	316	66.46	29042	virulence protein	RecName: Full=Flagellar motor switch protein FliM;
X566_24570	261	0	261	50.19	7743	virulence protein	flagellar basal body rod protein FlgG [Pseudomonas aeruginosa PAO1]
X566_24605	369	0	347	57.93	12262	virulence protein	RecName: Full=Flagellar P-ring protein; AltName: Full=Basal body P-ring protein; Flags: Precursor;
